# Identification of a Novel Polyomavirus from Patients with Acute Respiratory Tract Infections

**DOI:** 10.1371/journal.ppat.0030064

**Published:** 2007-05-04

**Authors:** Anne M Gaynor, Michael D Nissen, David M Whiley, Ian M Mackay, Stephen B Lambert, Guang Wu, Daniel C Brennan, Gregory A Storch, Theo P Sloots, David Wang

**Affiliations:** 1 Department of Molecular Microbiology, Washington University School of Medicine, St. Louis, Missouri, United States of America; 2 Queensland Paediatric Infectious Diseases Laboratory, Sir Albert Sakzewski Virus Research Centre, Royal Children's Hospital, Brisbane, Queensland, Australia; 3 Department of Internal Medicine, Washington University School of Medicine, St. Louis, Missouri, United States of America; 4 Department of Pediatrics, Washington University School of Medicine, St. Louis, Missouri, United States of America; 5 Department of Pathology and Immunology, Washington University School of Medicine, St. Louis, Missouri, United States of America; University of California San Francisco, United States of America

## Abstract

We report the identification of a novel polyomavirus present in respiratory secretions from human patients with symptoms of acute respiratory tract infection. The virus was initially detected in a nasopharyngeal aspirate from a 3-year-old child from Australia diagnosed with pneumonia. A random library was generated from nucleic acids extracted from the nasopharyngeal aspirate and analyzed by high throughput DNA sequencing. Multiple DNA fragments were cloned that possessed limited homology to known polyomaviruses. We subsequently sequenced the entire virus genome of 5,229 bp, henceforth referred to as WU virus, and found it to have genomic features characteristic of the family Polyomaviridae. The genome was predicted to encode small T antigen, large T antigen, and three capsid proteins: VP1, VP2, and VP3. Phylogenetic analysis clearly revealed that the WU virus was divergent from all known polyomaviruses. Screening of 2,135 patients with acute respiratory tract infections in Brisbane, Queensland, Australia, and St. Louis, Missouri, United States, using WU virus–specific PCR primers resulted in the detection of 43 additional specimens that contained WU virus. The presence of multiple instances of the virus in two continents suggests that this virus is geographically widespread in the human population and raises the possibility that the WU virus may be a human pathogen.

## Author Summary

We have identified a novel virus, referred to as WU virus, in the family Polyomaviridae by screening of human respiratory secretions. Two human polyomaviruses, BK and JC, were identified in 1971 and infect the majority of humans around the world. These two viruses are closely related to each other and are both are pathogenic in immunocompromised individuals. Earlier this year, a third polyomavirus, KI, was described in human clinical specimens, although its pathogenicity and prevalence in humans has not yet been established. The discovery of WU virus brings the number of polyomaviruses detected in humans to four. WU differs from BK and JC significantly in its genome sequence and in its relative tissue tropism, suggesting that it is likely to have unique biological properties. This discovery raises many questions for further investigation, such as, Is WU virus a human pathogen? If so, what kind of disease does it cause? Where in the body does WU virus reside? At what age does infection typically occur? Perhaps most importantly, there are likely to be many more as of yet unidentified viruses infecting the human body.

## Introduction

Viral infections of the respiratory tract are responsible for significant mortality and morbidity worldwide [1]. Despite extensive studies in the past decades that have identified a number of etiologic agents, including rhinoviruses, coronaviruses, influenzaviruses, parainfluenzaviruses, respiratory syncytial virus, and adenoviruses, approximately 30% of all cases cannot be attributed to these agents, suggesting that additional respiratory pathogens are likely to exist [2]. In fact, since 2001, six previously undescribed viruses have been identified by analysis of clinical specimens from the human respiratory tract: human metapneumovirus [3], SARS coronavirus [4], coronavirus NL63 [5], coronavirus HKU1 [6], human bocavirus [7], and the recently described KI virus [8]. In some instances, new molecular methods such as VIDISCA [5], pan-viral DNA microarrays [9], and high throughput sequencing [7,8] have played key roles in the identification of these agents. The advent of these new technologies has greatly stimulated efforts to identify novel viruses in the respiratory tract and in other human disease states.

Viruses in the family Polyomaviridae possess double-stranded DNA genomes and infect a variety of avian, rodent, and primate species. To date, two polyomaviruses, BK virus and JC virus, have been unambiguously described as human pathogens. BK and JC viruses are ubiquitous worldwide, and in adult populations, seroprevalence rates approaching 75% and 100%, respectively, have been reported [10]. Although human polyomaviruses have been suggested to utilize a respiratory route of transmission, detection of BK and JC polyomavirus nucleic acids in the respiratory tract has rarely been reported [11,12]. Infection with these two viruses is predominantly asymptomatic, although in the context of immunosuppression a number of syndromes have been clearly linked to these viruses. JC virus causes primary multifocal leukoencephalopathy, while BK virus has been associated with a variety of renal and urinary tract disorders, most importantly tubular nephritis, which can lead to allograft failure in renal transplant recipients and hemorrhagic cystitis in hematopoietic stem cell transplant recipients [13]. These viruses are believed to persist in a latent phase primarily in the kidney and can periodically undergo reactivation. Excretion of BK and JC viruses in urine has been reported in up to 20% of the general population [14,15]. Besides JC and BK virus, a very recent report has described a novel polyomavirus, KI, detected in human respiratory secretions and stool [8]. However, the pathogenicity and prevalence of this virus has not yet been established. In addition, in the late 1950s, ∼100 million people in the United States, and many more worldwide, may have been exposed to SV40, a polyomavirus that naturally infects rhesus monkeys via contaminated polio vaccines, leading to widespread debate about whether or not SV40 is capable of sustained infection and replication cycles in humans [16].

Much of the interest in polyomaviruses and SV40 in particular derives from the transforming properties carried by the early transcriptional region of the viral genome that encodes for the small T antigen (STAg) and and large T antigen (LTAg). T antigen is capable of binding both p53 and Rb proteins and interfering with their tumor suppressor functions. The early region alone is sufficient to transform established primary rodent cell lines [17] and in concert with telomerase and *ras* transforms primary human cells [18]. This has lead to controversy over whether any human tumors are associated with SV40 infection [19].

We describe the identification and characterization of a novel polyomavirus initially detected by high throughput sequencing of respiratory secretions from a patient suffering acute respiratory disease of unknown etiology. The virus was detected in the respiratory secretions from an additional 43 patients in two continents, and the complete genomes of multiple isolates were sequenced.

## Results

### Shotgun Sequencing of Respiratory Secretion

A nasopharyngeal aspirate (NPA) from a 3-year-old patient admitted to the pediatric ward of the Royal Children's Hospital in Brisbane with pneumonia was collected in October 2003. The patient had no other remarkable clinical traits other than the respiratory features of pneumonia. Testing of nucleic acid extracted from the NPA using a panel of 17 PCR assays for known respiratory viruses as described [20] yielded negative results. Total nucleic acid from the NPA was randomly amplified and cloned as described previously [9]. One 384-well plate of clones was sequenced using a universal M13 primer, and the resulting sequence reads were analyzed as described in Materials and Methods. Of the 384 reads, there were 37 poor quality sequences that were rejected from further analysis, 327 human sequences, six bacterial sequences, six viral sequences, and eight sequences of unknown origin that could not be classified. The bacterial sequences had greater than 97% nucleotide identity to known bacterial species, including Haemophilus influenzae (three reads), *Streptococcus pneumoniae, Corynebacterium pseudodiphthericum,* and Leifsonia xyli (unpublished data). Upon further examination, the six viral reads were collapsed into three unique regions, each of which possessed only limited homology to known polyomavirus proteins (sequences available in Figure S1). The highest scoring BLASTx hits for each of these three contigs possessed 35%, 50%, and 34% amino acid identity to JC virus STAg, BK virus LTAg, and SV40 VP1, respectively. At the time these experiments were performed, the KI virus genome had not yet been published. Subsequent analysis revealed amino acid identities of 66%, 65%, and 69% to KI virus for the three contigs. Furthermore, three of the eight previously unclassified sequence reads were determined to have between 58%–84% amino acid identity to KI virus VP1 and VP2 proteins by BLASTx analysis. Based on the limited sequence homology to known viruses, we tentatively assigned the name WU to the unknown polyomavirus.

### Complete Genome Sequencing and Genome Analysis

**Figure 1:**
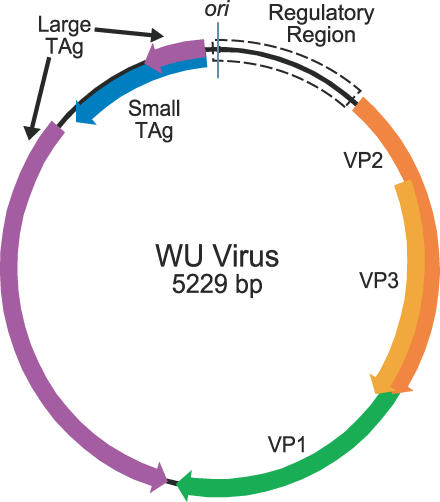
Schematic of WU Virus Genome Organization ori, origin of replication.

The complete genome of WU was sequenced to 3× coverage using cloned fragments of the viral genome generated by a series of PCR primers. Analysis of the DNA sequence revealed genomic features characteristic of polyomaviruses. First, the WU genome size of 5,229 base pairs (bp) was quite comparable to those of the primate polyomaviruses BK (5,153 bp), JC (5,130 bp), and SV40 (5,243 bp). In addition, the overall GC content of the WU genome was 39%, which is quite similar to the GC content of BK (39%), JC (40%), and SV40 (40%). The genome organization included an early region coding on one strand for STAg and LTAg, and a late region coding on the opposite strand for the capsid proteins VP1, VP2, and VP3 (Figure 1). These two regions were separated by a regulatory region that contained typical polyomavirus features. The regulatory region contained an AT-rich region on the late side of the putative replication origin. Three repeats of the consensus pentanucleotide LTAg binding site GAGGC were present, as was one copy of the non-consensus LTAg binding site TAGGC. While most polyomaviruses contain four copies of the consensus, baboon polyomavirus (simian agent 12) is a primate polyomavirus that contains only three copies of the canonical binding sequence and one non-consensus binding site [21]. Unusual features in the WU regulatory region included the presence of two partially overlapping LTAg binding sites and slightly variant spacing between the LTAg binding sites as compared to SV40, BK, and JC (Figure S2).

In the early region, an unspliced open reading frame of 194 amino acids was detected that possibly encodes for the STAg. As the paradigm in other polyomaviruses is that STAg is expressed from a spliced message, analysis of potential splice sites revealed the presence of a putative splice donor sequence just one nucleotide 5′ of the initially predicted stop codon. Splicing to a downstream putative splice acceptor site would excise an intron of 70 nucleotides and generate a slightly larger STAg of 217 amino acids (Figure S3). While the precise carboxyl terminus of the WU STAg has not yet been experimentally verified, sequence analysis revealed the presence of a highly conserved cysteine-rich motif, CX_5_CX_7–8_CXCX_2_CX_21–22_CSCX_2_CX_3_WF, that was present in both of the predicted isoforms of WU STAg. This motif, which is present in all STAgs, was perfectly conserved in WU virus with the exception of the initial cysteine residue.

In all polyomaviruses, the initial ∼80 amino acids of the N-terminus of the STAg and LTAg are identical; the LTAg is generated by alternative splicing of the early mRNA transcript. In WU virus, a conserved splice donor site was identified immediately after amino acid 84 of the early open reading frame. The position of the splice site is similar to that found in SV40, BK, and JC virus, which occur after amino acids 82, 81, and 81, respectively. Splicing to a conserved splice acceptor site would generate a predicted protein of 648 amino acids (Table 1). The predicted WU virus LTAg contained conserved features common to T antigens, including a DnaJ domain in the N terminus with the highly conserved hexapeptide motif HPDKGG; the LxCxE motif necessary for binding Rb; a canonical DNA binding domain; a zinc finger region; and conserved motifs GPXXXGKT and GXXXVNLE in the ATPase-p53 binding domain [22].

**Table 1:**
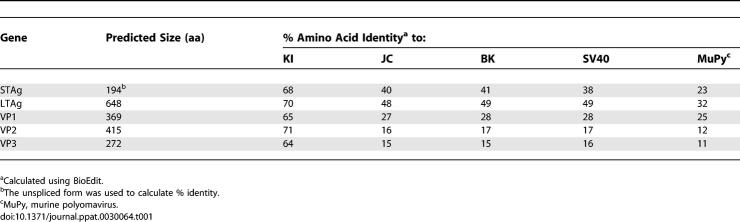
Homology of Predicted WU Proteins

Based on comparative sequence analysis of LTAgs, the polyomaviruses are classified into two subclasses: a primate-like group exemplified by SV40, and a mouse polyoma-like group exemplified by murine polyoma virus [22]. Using these criteria, the T antigen of WU appeared to more closely resemble the mouse polyoma-like class of virus than the primate class. First, the mouse polyoma-like viruses have insertions of varying length after amino acids 66 and 113 of SV40 as compared to the primate class. In the amino terminal domain of the WU virus LTAg, multiple sequence alignment revealed the presence of a two–amino acid and a ten–amino acid insertion at these two loci, respectively. Furthermore, the primate-like class typically contains an extension of the carboxyl terminus termed the host range domain that is absent in the mouse polyoma-like class. In contrast to SV40, BK, JC, and baboon polyomavirus, WU virus did not appear to encode a carboxyl terminal extension (Figure S4).

In addition to encoding LTAg and STAg, murine and hamster polyomaviruses utilize alternative splicing to generate an intermediate-sized protein referred to as middle T antigen. The WU virus early region was scanned for splicing motifs similar to known murine and hamster polyomavirus splice donor and acceptor sequences, but no obvious combination of splice sites was detected that would yield a middle T antigen sequence in the size range of known middle T antigens. In addition, SV40, JC, BK, and baboon polyomavirus all encode a fourth late protein termed the agnoprotein. There was no open reading frame present in WU with any detectable homology to the known agnoproteins. Thus, our sequence analysis suggests that neither middle T antigen nor agnoprotein are encoded by WU virus, although it is possible that the sequences have diverged beyond our ability to recognize the appropriate splice sites or protein products.

### Phylogenetic Analysis

**Figure 2:**
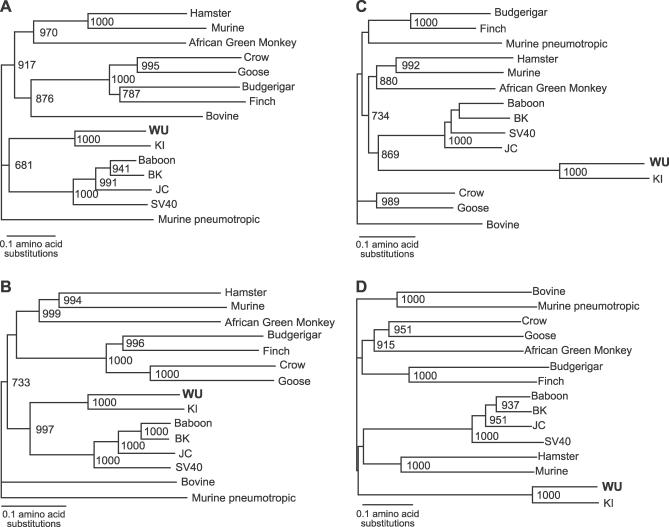
Phylogenetic Analysis of WU Virus Amino acid–based phylogenetic trees were generated using the neighbor-joining method with 1,000 bootstrap replicates. Significant bootstrap values are shown. A) STAg; B) LTAg; C) VP1; D) VP2.

Multiple sequence alignments of the predicted STAg, LTAg, VP1, and VP2 open reading frames revealed that WU virus was clearly a novel virus that is most closely related to KI virus (Figure 2). Neighbor-joining analysis suggested that these two viruses appear to form a new subclass of polyomaviruses. In the early region and VP1 protein, the WU/KI branch was most closely related to the known primate polyomaviruses BK, SV40, JC, and baboon polyomavirus (Figure 2A–2C). Finally, the VP2 open reading frame was so divergent that its evolutionary relationship to other polyomaviruses aside from KI could not be reliably established (Figure 2D). Analysis of the VP3 amino acid sequence, which is completely contained within VP2, gave similar results as VP2 (unpublished data).

### Prevalence of WU

PCR primers were designed to specifically amplify WU. The initial screen used primers targeting the VP2 region, which possessed less than 20% amino acid homology to JC and BK virus to minimize the possibility of cross reactivity with the known human polyomaviruses. Empirical testing of the primers on samples known to contain BK and JC confirmed that the primers did not cross react with either of these genomes (unpublished data). Positives in the initial screen for WU virus were sequenced and then further confirmed by a second PCR reaction using primers targeting the 3′ end of the WU virus LTAg coding sequence. All 43 positive samples in the initial screen were confirmed using the second pair of PCR primers. A subset of samples that tested negative in the initial screen was also tested with the second PCR primer pair, and none of those samples were positive.

### Brisbane, Queensland, Australia, Cohort

In order to assess the prevalence of WU polyomavirus, a cohort of 1,245 respiratory specimens collected in 2003 in Brisbane was examined. Thirty-seven out of the 1,245 (3.0%) samples tested were positive for the virus (Table 2). In this cohort, patients that tested positive ranged in age from 4 months to 53 years. The vast majority of the patients (33/37) were age 3 and under. In 12 patients with clear clinical evidence of respiratory tract infection, WU was the sole virus detected. Strikingly, in 25 of the 37 positive samples, one or more additional respiratory viruses were also detected. The most common co-infections were with rhinovirus (15 cases) and human bocavirus (ten cases). Furthermore, in one sample, a total of four viruses (WU, bocavirus, rhinovirus, and adenovirus) were detected, and in six other samples, a total of three viruses were detected (Table 2).

**Table 2:**
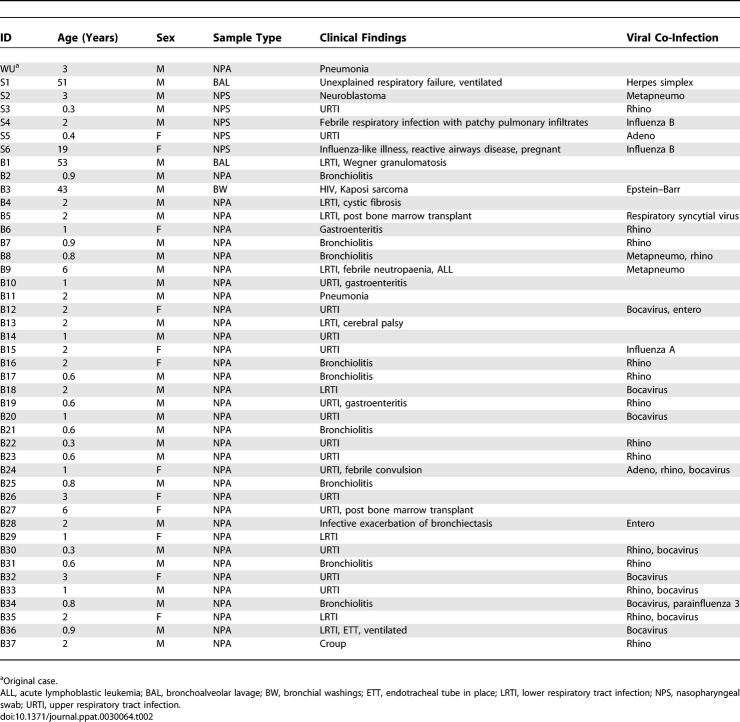
Patients Positive for WU Virus

### St. Louis, Missouri, United States, Cohorts

In addition, we examined two cohorts of patients from St. Louis, Missouri, United States. In one set of upper respiratory specimens collected in 2006, five out of 410 were positive for WU virus in the PCR assay. In addition, 480 bronchoalveolar lavage samples from patients (mostly adults) with severe acute respiratory illness were tested, yielding one positive. Of the positive samples, all six were co-infected with other viruses (Table 2). The age range of the positive cases varied from 4 months to 51 years.

### Strain Variants

**Figure 3:**
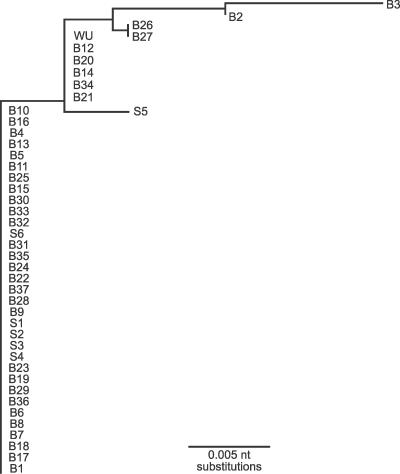
Strain Variation of WU virus A 250-bp fragment of the VP2 gene was aligned using ClustalX. WU indicates the original case, and strain designations correspond to patients as listed in Table 2.

To assess the sequence variation within different isolates, we analyzed the 250-bp region encompassed by the initial screening primers for all 43 cases (Figure 3). Several divergent strains were detected, including one sample that had five mutations (2%) within this region. In another case, a 12-bp deletion was observed. The fact that many isolates were identical in sequence was not surprising, given the relatively short length of the amplicon and the double-stranded DNA nature of the genome. In addition, we sequenced the complete genome of five additional isolates from five independent patients. Unfortunately, efforts to completely sequence the two most divergent isolates (based on the 250-bp sequence, B2 and B3) have been unsuccessful, presumably due to low viral titers in these samples. All six complete genomes were 5,229 bp in size, and overall, there was between 0.08% and 0.23% sequence variation from sample to sample, well above that expected from Taq PCR, ruling out the possibility that the additional positives were artifacts of PCR contamination. Moreover, the majority of the observed mutations were synonymous substitutions or in non-coding regions, lending further support to the argument that these were authentic strain variants. For JC virus, the reported intratype sequence variation is of a similar magnitude, ranging between 0.1% and 0.5% [23].

### Screening of Urine

Because BK and JC virus are frequently excreted in urine, we examined urine samples from patient cohorts in both St. Louis and Brisbane for the presence of WU virus by PCR. In the St. Louis cohort, urine samples from 200 adult patients participating in a study of polyomavirus infections in kidney transplant recipients were tested [24]. For most patients, samples were tested at three time points: prior to transplant, 1 mo post transplant, and 4 mo post transplant, although for some patients the pre-transplant specimen was not available. Zero out of 501 samples tested were positive for the WU polyomavirus. As a control, using previously validated BK primers, we were able to amplify BK virus in a subset of these urine samples, confirming the integrity of the specimens themselves (unpublished data). Similarly, from the Brisbane cohort, none of the 226 urine samples tested were positive for WU virus.

## Discussion

We used a high throughput sequencing strategy to search for novel agents that were present in respiratory tract infections of unknown etiology. The focus of this study was on individual clinical specimens that still lacked a diagnosis after analysis with an extensive panel of diagnostic assays for known respiratory viruses. In one such patient sample, novel sequences with limited homology to known polyomaviruses were detected. Complete genome sequencing and phylogenetic analysis revealed that the new virus clearly had the genomic organization typical of polyomaviruses but was divergent from all previously described polyomaviruses. In keeping with the two-letter virus names for human polyomaviruses, we have named this novel polyomavirus WU virus [25,26]. Overall, the predicted amino acid sequences of WU virus proteins were most similar to the newly described KI virus (Table 1). Outside of KI, WU shared only ∼15%–49% identity to its closest relatives (Table 1).

Detailed analysis of the viral DNA sequence and genomic organization confirmed the novelty of WU virus. At all loci, WU virus was most similar to KI virus, but the degree of divergence between WU and KI was greater than the divergence between SV40 and BK, indicating that WU and KI are clearly distinct viruses (Figure 2). Based on the phylogenetic analysis, it appears that WU and KI define a novel branch within the Polyomaviridae family (Figure 2). Relative to the established polyomaviruses, some analyses suggested that the WU/KI branch might be more closely related to the primate polyomaviruses, while other features of the WU genome suggested that it might be more similar to murine polyomavirus. For example, neighbor-joining phylogenetic analysis suggested that the predicted STAg, LTAg, and VP1 open reading frames of both KI and WU were most closely related to SV40, JC, BK, and baboon polyomaviruses. Analysis of the VP2/VP3 region was more equivocal, as the proteins were too divergent to reliably assess. The apparent absence of the C-terminal “host range” domain in the LTAg and the agnoprotein open reading frame, both of which are present in the known primate polyomaviruses, suggested that WU virus was more similar to murine polyomavirus than the primate polyomaviruses by these criteria. While the evolutionary history of this virus is not clear at the moment, the totality of the analysis indicates that WU is clearly a unique virus.

We detected WU in 37 out of 1,245 (3.0%) patient specimens in Brisbane (excluding the original case) and in six out of 890 (0.7%) patient specimens tested in St. Louis. As the positive specimens were all collected from 2003 through 2006, it appears that WU is currently circulating, and its presence in both North America and Australia suggests that the virus is geographically widespread in the human population. The age range of patients that tested positive for WU virus spanned from 4 months to 53 years. The majority (86%) of the cases were found in children 3 years of age and under. Of the four positive specimens from adult patients (S1, S6, B1, and B3 in Table 2), three clearly had altered immune status. One patient was HIV-positive, one was immunosuppressed due to treatment for Wegener granulomatosis, and one was pregnant. The fourth adult patient (S1), while not obviously immunosuppressed, also suffered from liver cirrhosis, hypertension, type 2 diabetes, and co-infection with herpes simplex virus, and required mechanical ventilation. In addition, there were two other positive patients older than 3 years of age: a 6-year-old child who had previously been a bone marrow transplant recipient (Table 2, B27) and a 6-year-old child diagnosed with acute lymphoblastic leukemia (Table 2, B9). While preliminary, the age distribution of the positive cases in this study combined with the established paradigms for BK and JC virus suggest a model where acute infection with WU virus may occur relatively early in life and result in a latent infection. Immunosuppression or other insults such as viral infection could then lead to reactivation of WU virus in older individuals.

The patients who yielded positive specimens suffered from a wide range of respiratory syndromes, including bronchiolitis, croup, and pneumonia as well as other clinical maladies (Table 2). Detection of WU virus sequences in these patients is merely the first step in assessing the potential etiologic role of WU virus in acute respiratory tract disease. It is not yet known whether WU is infectious or whether it is capable of replication in the respiratory tract. One possibility is that WU is not involved at all in respiratory disease, but rather is simply transmitted by the respiratory route. The human polyomaviruses BK and JC are hypothesized to be transmitted by the respiratory route before taking up residency primarily in the kidneys. Latency in the kidneys of BK and JC is believed to be the reason that both viruses are excreted in the urine of up to 20% of asymptomatic individuals [14,15]. In this study, using the same PCR assays that were effective in respiratory secretions, we did not detect WU in any of the 727 urine samples we tested. The lack of detection of WU virus in the urine may reflect sensitivity issues, a bias in the cohorts tested, or simply that WU is unlike BK and JC viruses and is not secreted in the urine. A similar tissue profile to that of WU virus has been reported in initial studies of KI virus [8]. Future experiments will aim to determine the tissue tropism of WU and whether any tissue reservoirs for WU virus exist.

In the literature, there is one animal polyomavirus that has been found extensively in lung tissue. Infection of suckling mice with the mouse pneumotropic polyomavirus (MPPV) causes interstitial pneumonia and significant mortality. MPPV also differs from other polyomaviruses in that besides the kidneys, it can also be detected in the lungs, liver, spleen, and blood of suckling mice [27]. Thus, there is precedence for an animal polyomavirus causing respiratory disease, suggesting at least the possibility that WU virus could be similarly pathogenic in humans.

One striking observation from these studies is the relatively high frequency of co-infection detected in the respiratory secretions: 72% overall (100% in the St. Louis cohort and 68% in the Brisbane cohort). Although more extensive studies are necessary to confirm the generality of this observation, this raises several intriguing non-mutually exclusive possibilities to consider: 1) WU may be an opportunistic pathogen; 2) WU infection may predispose or facilitate secondary infection by other respiratory viruses; and 3) WU may be a part of the endogenous viral flora that is reactivated by inflammation or some other aspect of viral infection. Recent studies of the prevalence of the newly identified human bocavirus have also reported higher levels of co-infection than previously described for other viruses found in the respiratory tract, with co-infection rates as high as 50% reported [28,29]. In addition, five of six samples positive for KI virus were reported to be co-infected with other known respiratory viruses [8]. As detection methods improve in sensitivity and more comprehensive efforts are made to examine the diversity of viruses found in the respiratory tract, a greater appreciation for the rates of dual or multi-infection is gradually emerging. For example, the use of extensive panels of PCR assays in this study revealed that one of the positive specimens was quadruply infected; adenovirus, rhinovirus, and bocavirus and WU virus were all present. Further investigations that aim to systematically define the spectrum of viruses present in the respiratory tract are clearly warranted so that the possible roles that co-infections may play in disease pathogenesis can be explored.

Extremely high sequence divergence was observed in the capsid proteins VP1 and VP2 of WU virus and KI virus as compared to the other known polyomaviruses. This divergence may reflect a different “lifestyle” for the WU/KI branch as compared to known polyomaviruses. Our data demonstrating the presence of WU in respiratory secretions and its absence in urine samples suggest that the mode of transmission or the sites of persistence of WU may be distinct from the other human polyomaviruses. As such, the structure of the virion must be optimized to enable the virus to survive dramatically distinct physiological and environmental conditions. This may partially explain the observed sequence divergence in the capsid proteins.

Another question raised by this study relates to the potential antigenic cross reactivity of the WU capsid proteins. In terms of establishing the seroprevalence of WU itself and determining whether seroconversion accompanies acute infection with WU, it will be essential to conduct these studies with consideration for potential cross reactivity to KI, BK, JC, and SV40 antibodies. In addition, it is tantalizing to speculate whether serum antibodies to WU have the potential to cross react to SV40-derived antigens, and if so, whether they may at least partially account for some of the studies that report the presence of SV40 antibodies in the human population that is too young to have suffered exposure from contaminated polio vaccination [30–32].

In conclusion, we have identified and completely sequenced the genome of a novel polyomavirus. This virus appears to be geographically widespread in the human population as evidenced by the detection of 44 distinct cases in two continents. Based on preliminary analysis, WU and KI virus share some strikingly similar properties, including their complement of genes, phylogenetic relationship, and physical sites of detection in the human body. These data suggest that WU virus and KI virus define a novel branch within the Polyomaviridae family with unexplored biology and pathogenicity. Another implication of these results is that the diversity of viruses in this family may be far greater than currently realized. Further experimentation is now underway to determine the relative pathogenicity of WU virus in humans and to understand the molecular properties of the virus. Since the T antigen of WU is predicted to have transforming properties by analogy to other polyomavirus T antigens, one question currently under investigation is whether a subset of human tumors may be associated with WU.

## Materials and Methods

### Clinical specimens—Respiratory secretions.

Brisbane cohort. A total of 1,245 specimens (predominantly NPAs) were collected between January 1, 2003, and December 22, 2003, from patients presenting to the Royal Children's Hospital in Brisbane, Queensland, Australia, with symptoms consistent with acute lower respiratory tract infection.

St. Louis cohort #1. A total of 480 BAL specimens were tested. These included samples from a retrospective and a prospective collection. The retrospective specimens were from a sequential collection of BAL specimens submitted routinely to the Virology Laboratory at St. Louis Children's Hospital between December 2002 and August 2003 [33]. For the present study, an effort was made to select specimens from this collection from patients with acute respiratory illness, and to exclude specimens collected as routine post–lung transplant surveillance. The prospective specimens were from an ongoing study of the etiology of severe acute respiratory illness and were collected between October 2005 and October 2006. Both collections included specimens from patients of all ages, although the large majority were from adults.

St. Louis cohort #2. This collection was made up of respiratory specimens, mostly nasopharyngeal swabs, submitted for routine virologic testing to the Virology Laboratory at St. Louis Children's Hospital between September 2005 and June 2006. The majority of these specimens were from children. Of the 410 specimens in this collection, 200 were selected because they had been found to be positive by fluorescent antibody staining or culture for influenzavirus A or B, respiratory syncytial virus, parainfluenza virus, rhinovirus, or adenovirus.

### Clinical specimens—Urine.

Brisbane cohort. Urine specimens (226) that were submitted during 2003 to the diagnostic laboratory for routine investigation were collected. These represented a diverse mixture of donors, including those from (i) sexual health clinic (*n* = 50), (ii) pediatric clinic (*n* = 52), (iii) antenatal clinic (*n* = 33), (iv) indigenous health clinic (*n* = 36), and (v) bone marrow transplant patients (*n* = 55).

The St. Louis urine specimens were from a study of polyomaviruses in adult renal transplant recipients [24]. A total of 200 individuals were enrolled in the study between December 2000 and October 2002. From each patient, up to three specimens were tested, including a specimen obtained before the transplant and specimens obtained at 1 and 4 mo after transplantation.

### Diagnostic testing of clinical specimens for known respiratory viruses.

Brisbane cohort. Nucleic acids were extracted from 0.2 ml of each specimen using the High Pure Viral Nucleic Acid kit (Roche Diagnostics Australia, http://www.rochediagnostics.com.au) according to the manufacturer's instructions. PCR assays for 17 known respiratory viruses were performed as described [20].

St. Louis cohort. All respiratory specimens were tested originally by fluorescent antibody staining using a panel of monclonal antibodies directed against influenza A and B, respiratory syncytial, parainfluenza 1–3, and adenoviruses (Simulfluor Respiratory Screen; Chemicon, http://www.chemicon.com). Specimens that were negative were also cultured using cell culture systems that could detect the same group of viruses plus rhinoviruses, cytomegalovirus, and herpes simplex virus. Total nucleic acid extracts were purified using a Qiagen M48 instrument (http://www.qiagen.com). Nucleic acid extracts were tested for a panel of respiratory viruses using the EraGen MultiCode-PLx respiratory virus panel (EraGen Biosciences, http://www.eragen.com), a multiplex PCR assay that detects the following viruses: influenza A and B, respiratory syncytial virus A and B, parainfluenza 1–4, human meatpneumovirus, adenovirus subgroups B, C, and E, rhinoviruses, and coronaviruses OC43, 229E, and NL63.

### Library construction and shotgun sequencing.

Samples were prepared in the following manner for high throughput sequencing analysis. A total of 200 ul of neat NPA sample was thawed and directly treated with DNase I (Fermentas, http://www.fermentas.com) for 60 min at 37 °C. Total nucleic acid was extracted using the Masterpure Complete DNA and RNA Purification Kit (Epicentre Biotechnologies, http://www.epibio.com). Then, 100 ng of total nucleic acid was randomly amplified using the RdAB protocol exactly as described [9]. RNA in the total nucleic acid preparation was converted to cDNA by reverse transcription with primer-A (5′ GTTTCCCAGTCACGATANNNNNNNNN). Two rounds of random priming with primer-A and extension with Sequenase (United States Biochemical, http://www.usbweb.com) enabled second strand cDNA synthesis as well as random priming of DNA originally present in the total nucleic acid sample. Amplicons were then generated via 40 cycles of PCR using primer-B (5′ GTTTCCCAGTCACGATA) with a cycling profile of: 94 °C 30 s; 40 °C 30 s; 50 °C 30 s; 72 °C 60 s. The primer-B–amplified material was TOPO cloned into pCR4.0 (Invitrogen, http://www.invitrogen.com) and transformed into bacteria, and white colonies were picked into 384-well plates. DNA was purified by magnetic bead isolation and sequenced using standard Big Dye terminator (v3.1) sequencing chemistry. Reaction products were ethanol precipitated, resuspended in 25 ul of water, and loaded onto the ABI 3730xl sequencer.

### Analysis of shotgun sequences.

Sequences were assessed for quality using Phred [34], and reads that contained less than 50 contiguous bases with a score of phred 20 or greater were rejected. The remaining reads were analyzed in the following steps: 1) reads were aligned to the human genome using BLASTn with an e^−10^ cutoff; 2) remaining reads were aligned to a bacterial database using BLASTn with an e^−10^ cutoff; and 3) remaining reads were aligned to the viral RefSeq protein database using BLASTx with an e^−2^ cutoff [35].

### Complete genome amplification and sequencing.

The WU genome derived from the index case was sequenced to 3× coverage using six unique pairs of PCR primers for the amplification. Amplicons were cloned into pCR4.0 and sequenced using standard sequencing technology. All primers used for amplification and sequencing are listed in Table S1 and their positions depicted in Figure S5. Additional complete genomes were sequenced to at least 2× coverage using the same primers listed in Table S1. Completed genome sequences have been deposited into GenBank (see Supporting Information for accession numbers).

### Phylogenetic analysis.

Protein sequences associated with the following reference virus genomes were obtained from GenBank: BK virus, JC virus, bovine polyomavirus, SV40, baboon polyomavirus (simian agent 12), finch polyomavirus, crow polyomavirus, goose hemorrhagic polyomavirus, African green monkey polyomavirus, budgerigar fledgling polyomavirus, murine pneumotropic virus, hamster polyomavirus, and murine polyomavirus (see Supporting Information for accession numbers). For WU virus, predicted open reading frames were used. For STAg, the predicted open reading frame of 194 amino acids was used for analysis. Multiple sequence alignment was performed using ClustalX (1.83). Neighbor-joining trees were generated using 1,000 bootstrap replicates.

### Nucleic acid prevalence studies.

For all PCR assays, standard precautions to avoid end product contamination were taken, including the use of PCR hoods and maintaining separate areas for PCR set up and analysis. For initial screening of WU virus, PCR primers AG0044 5′ tgttacaaatagctgcaggtcaa and AG0045 5′ gctgcataatggggagtacc were used with Accuprime hot start Taq (Invitrogen) to amplify 1 ul of template using the following program: 40 cycles of 94 °C 30 s; 56 °C 30 s; 72 °C 60 s. For every 88 samples tested, seven no-template negative controls were interspersed between the actual samples. Products were visualized following electrophoresis on 1% agarose gels. The resulting 250-bp amplicon was sequenced directly in both directions using primer AG0044 and AG0045. These sequences have been deposited in GenBank (see Supporting Information for accession numbers). Secondary confirmation was performed using primers AG0048 5′ TGTTTTTCAAGTATGTTGCATCC and AG0049 5′ CACCCAAAAGACACTTAAAAGAAA that generate a 244-bp amplicon in the 3′ end of the LTAg coding region. The same cycling profile of 40 cycles of 94 °C 30 s; 56 °C 30 s; 72 °C 60 s was used. For detection of both BK and JC viruses, primers AG0068 5′ AGTCTTTAGGGTCTTCTACC and AG0069 5′ GGTGCCAACCTATGGAACAG were used with a profile of 40 cycles of 94 °C 30 s; 56 °C 30 s; 72 °C 60 s.

## Supporting Information

Figure S1 Raw Sequence Data from High Throughput Screening A) The initial six shotgun reads with homology to polyomaviruses. B) The three contigs derived from the six reads.
(38 KB PDF)

Figure S2 Comparison of SV40 and WU Virus Replication Origin Region The consensus TAg binding motif is GAGGC. The known primate polyomaviruses SV40, JC, BK, and baboon polyomavirus all have four copies of the copies of the binding site oriented as shown above for SV40 (NC_001669). The first nucleotide of the third copy of the consensus TAg binding site is defined as nucleotide 1 for WU and SV40. Differences between SV40 and WU Virus are 1) one of the TAg binding sites in WU virus appears to be a non-canonical TAGGC; 2) the second and third consensus TAg binding sites in WU virus overlap; and 3) the nucletoide spacing between the TAg binding sites in WU virus varies from the prototype SV40 as shown. Shown in blue is the polyA/T tract that is commonly found to the late side of the origin in polyomaviruses
(462 KB PDF)

Figure S3 Predicted Splice Sites for LTAg and STAg A consensus LTAg donor site was detected. Splicing to the consensus downstream acceptor would generate a LTAg of 648 amino acids. For STAg, an unspliced open reading frame of 194 amino acids was identified. A predicted slice donor site was also detected that would result in excision of a 70-nucleotide intron and production of a 217–amino acid open reading frame.
(542 KB PDF)

Figure S4 WU Virus Lacks a Carboxyl Terminus Extension of the the LTAg Multiple sequence alignment of WU virus LTAg with 13 other reference sequences reveal the presence of carboxyl terminus extensions in baboon polyoma, BK, JC, and SV40. WU virus does not appear to encode such a region.
(5.4 MB PDF)

Figure S5 Map of Primers and Sequence Reads Locations of original shotgun reads are depicted as indicated. Locations of all sequencing primers are mapped to the complete genome. Primers used for amplification are shown in red.
(551 KB PDF)

Table S1 Primers Used for Amplification and Sequencing of WU Virus (35 KB PDF)

### Accession Numbers

The GenBank (http://www.ncbi.nlm.nih.gov/Genbank) protein sequences used in this paper are as follows:

LTAg: African green monkey (NP_848008); baboon polyomavirus 1 (YP_406555); BK (YP_717940); bovine (NP_040788); budgerigar (NP_848014); crow (YP_529828); finch (YP_529834); goose (NP_849170); hamster (NP_056730); JC (NP_043512); KI Stockholm 60 (ABN09921); murine (NP_041264); murine pneumotropic (NP_041232); SV40 (NP_043127).

STAg: African green monkey (NP_848009); baboon polyomavirus 1 (YP_406556); BK (YP_717941); bovine (NP_040789); budgerigar (NP_848015); crow (YP_529829); finch (YP_529835); goose (NP_849171); hamster (NP_056732); JC (NP_043513); KI Stockholm 60 (ABN09920); murine (NP_041266); murine pneumotropic (NP_041233); SV40 (NP_043128).

VP1: African green monkey (NP_848007); baboon polyomavirus 1 (YP_406554); BK (YP_717939); bovine (NP_040787); budgerigar (NP_848013); crow (YP_529827); finch (YP_529833); goose (NP_849169); hamster (NP_056733); JC (NP_043511); KI Stockholm 60 (ABN09917); murine (NP_041267); murine pneumotropic (NP_041234); SV40 (NP_043126).

VP2: African green monkey (NP_848005); baboon polyomavirus 1 (YP_406552); BK (YP_717937); bovine (NP_040785); budgerigar (NP_848011); crow (YP_529825); finch (YP_529831); goose (NP_849167); hamster (NP_056734); JC (NP_043509); KI Stockholm 60 (ABN09918); murine (NP_041268); murine pneumotropic (NP_041235); SV40 (NP_043124).

WU complete genome sequences have been deposited under accession numbers EF444549–EF444554. VP2 partial sequences have been deposited under accession numbers EF444555–EF444593.

## Abbreviations

bp: base pair
LTAg: large T antigen
NPA: nasopharyngeal aspirate
STAg: small T antigen
