# Supplementary material for: Identification of a Novel Polyomavirus from Patients with Acute Respiratory Tract Infections
Source: PLoS Pathog. 2007 May 4;3(5):e64. doi: 10.1371/journal.ppat.0030064 (PMC1864993; doi:10.1371/journal.ppat.0030064)
Supplement: Supplementary file 1 [file ppat.0030064.sg001.pdf]

A

```
>DWAB-aaa05a03.g1
GCACTTATAGCAGTAACACTCTATCCACATTGGAGGTTTTCTAAATATTTTATATTTTTGCTTGTGGCGCT
TGTCTAAAAAGCAGTAAAAACAATTACACAAATAATAAAACCCCTTTAAGCATATGTCCCAGTCTTTTAAA
ATATACTCTTCAAAAACATCACCATAAACTTCTCCAACAAGCCTGTACTTTCTAGGGGGAAAGTTACAGCA
CAATTCTGTGCATTCTACCTGTGAAGAACTCCACACTTCATCTTCTTCTTCATTTAGTTGGTGCACTGTAC
TAACACACTCTTGCAGTTTTAAATATAAAGAATTAAGCTTTTTCATTTTTTCCTCATTTCCCCCTTTGTCA
GG
>DWAB-aaa05a04.g1
GCAGCTGTAGCAGTAACACTCTATCCACATTGGAGGTTTTCTAAATATTTTATATTTTTGCTTGTGGCGCT
TGTCTAAAAAGCAGTAAAAACAATTACACAAATAATAAAACCCCTTTAAGCATATGTCCCAGTCTTTTAAA
ATATACTCTTCAAAAACATCACCATAAACTTCTCCAACAAGCCTGTACTTTCTAGGGGGAAAGTTACAGCA
CAATTCTGTGCATTCTACCTGTGAAGAGCTCCACACTTCATCTTCTTCTTCATTTAGTTGGTGCACTGTAC
TAACACACTCTTGCAGTTTTAAATATAAAGAATTAAGCTTTTTCATTTTTTCCTCATTTCCCCCTTTGTCA
GG
>DWAB-aaa05h07.g1
TGGCTTATTAAACACCTGCTTATAAAGCAAATTTATAGTATATGGGTTTCTAACCCTTCTTTGTCTAAAGT
GTAGCCTAAAAAATCTGGAAAGCCCTGTATGTACTCTATTTTTGTTAACTCCCAAAAGGTCAGCACAAGTT
ATGTACAATCTACCTTGCAAGCAAAGAATGCCAATGCCATTTTCATCCAACAGTGGAATTGTGCTATTATT
ACTAAATGACACCACTGGTGGAGTTGCAGCCCCTCCAACCATTCTGCCAAAGTATCTGCAGTTATCATTGC
GGCTAGGGTCTGCCACCCATGATTCAATGCTGTACTTTTCATTAGTGACCCTTGCCCTGGGTGTTTCAGAA
CTGGAATAAACATGAGGCACAGTACCATTGGTTTTAGGATTTACAGTATACTGAG
>DWAB-aaa06g03.g1
GTTGACACACATTTTTTTGACTTTTACTGTCTGAAAATAGTAAAGCATTTTTTTGATGTTCCATATGAAGT
CTATTATGAGTTGCATCTTCATTACTATTGCATTTTTCACACTCTTCTACCTTAATAGATAGTTGTAAATA
TAATCCAAGTAATAAGTACACATCATCAATTCCTAATTCTAAAGCAAATTCTGACAAAGCCTTCCAATTTA
ATTGATCTTTAAACTCCCCATATAAATCCTCAGCTTTAAAATCATTTTCTTTTAAGCCACCAGGAATATTT
TCTTCACATAAAGTAAATGGATCTCTAGTCATTCTACTATATAAACCATATGCATTATTAACACCTTTACA
AAATAAAAAGCTAATAGTACAGTATCCCTTACAAAAGTTATTAACAGCACTAACTCTATGTCTAAAAGGTG
TTAAAATAAACACAAGTGCAGTATTATAATAAGAATGTCTACTTGCAAAATTACATTTAAACTTACTTAAA
AGTTTTTTATATAGGGTTTCTGCCTTTTCTTTGGTGGTATGTATTACAAATGCAGTTAAAGTTCTATTACT
AAATACAGCTTGAGACACAAATTCTTCCAATTCTTTAGGAAATGATAAAGATGCATCTGTAGCATTGTCCT
TTTTTTTTTT
>DWAB-aaa06h01.g1
TTCTAACCCTTCTTTGTCTAAAGTGTAGCCTAAAAAATCTGGAAAGCCCTGTATGTACCCTATTTTTGTTA
ACTCCCAAAAGGTCAGCACAAGTTATGTACAATCTACCTTGCAAGCAAAGAATGCCAATGCCATTTTCATC
CAACAGTGGAATTGTGCTATTATTACTAAATGACACCACTGGTGGAGTTGCAGCCCCTCCAACCATTCTGC
CAAAGTATCTGCAGTTATCATTGCGGCTAGGGTCTGCCACCCATGATTCAATGCTGTACTTTTCATTAGTG
ACCCTTGCCCTGGGTGTTTCAGAACTGGAATAAACATGAGGCACAGTACCATTGGTTTTAGGATTTACAGT
ATACTGAGCAGGCCCCTTGTATTTTTCAGTGGGAGCAAGTCCTAGCACATCCAAGGGCTGTCCTCCAACTG
CCCAAAAATAAAGTTGGGGACCAGCAATACCTCCTACACCCCCTGTAGTAAGTATGCCAGCATTAAGCACA
GGAAGCACCAAAACTTCTGTTTCCATTCTATACAGCTCCCATATAAGCATGTCACATTCACTAACCTGATT
AGGAATATCAGGGGGAGCAACCTGTGACAAGCTGTAGCACACAGTAGTATCGGCATCAGGAGTAACATTAG
CAGCTTCAGCAGTTTTAAGTGGGCTACTAATAGACCAATAATGTGGGGTAGTCCCCTCTGCATTTCCAATA
ACAGGTTTAACAAACAATTCAA
>DWAB-aaa08f04.g1
TCCCCCTGATATTCCTAATCAGGTTAGTGAATGTGACATGCTTATATGGGAGCTGTATAGAATGGAAACAG
AAGTTTTGGTGCTTCCTGTGCTTAATGCTGGCATACTTACTACAGGGGGTGTAGGAGGTATTGCTGGTCCC
CAACTTTATTTTTGGGCAGTTGGAGGACAGCCCTTGGATGTGCTAGGACTTGCTCCCACTGAAAAATACAA
GGGGCCTGCTCAGTATACTGTAAATCCTAAAACCAATGGTACTGTGCCTCATGTTTATTCCAGTTCTGAAA
CACCCAGGGCAAGGGTCACTAATGAAAAGTACAGCATTGAATCATGGGTGGCAGACCCTAGCCGCAATGAT
AACTGCAGATACTTTGGCAGAATGGTTGGAGGGGCTGCAACTCCACCAGTGGTGTCATTTAGTAATAATAG
CACAATTCCACTGTTGGATGAAAATGGCATTGGCATTCTTTGCTTGCAAGGTAGATTGTACATAACTTGTG
CTGACCTTTT
```

B

>ContigA(05a03.05a04)
GCACTTATAGCAGTAACACTCTATCCACATTGGAGGTTTTCTAAATATTTTATATTTTTG
CTTGTGGCGCTTGTCTAAAAAGCAGTAAAAACAATTACACAAATAATAAAACCCCTTTAA
GCATATGTCCCAGTCTTTTAAAATATACTCTTCAAAAACATCACCATAAACTTCTCCAAC
AAGCCTGTACTTTCTAGGGGGAAAGTTACAGCACAATTCTGTGCATTCTACCTGTGAAGA
ACTCCACACTTCATCTTCTTCTTCATTTAGTTGGTGCACTGTACTAACACACTCTTGCAG
TTTTAAATATAAAGAATTAAGCTTTTTCATTTTTTCCTCATTTCCCCCTTTGTCAGG
>ContigB (06g03)
GTTGACACACATTTTTTTGACTTTTACTGTCTGAAAATAGTAAAGCATTTTTTTGATGTTC
CATATGAAGTCTATTATGAGTTGCATCTTCATTACTATTGCATTTTTCACACTCTTCTACC
TTAATAGATAGTTGTAAATATAATCCAAGTAATAAGTACACATCATCAATTCCTAATTCTA
AAGCAAATTCTGACAAAGCCTTCCAATTTAATTGATCTTTAAACTCCCCATATAAATCCTC
AGCTTTAAAATCATTTTCTTTTAAGCCACCAGGAATATTTTCTTCACATAAAGTAAATGGA
TCTCTAGTCATTCTACTATATAAACCATATGCATTATTAACACCTTTACAAAATAAAAAGC
TAATAGTACAGTATCCCTTACAAAAGTTATTAACAGCACTAACTCTATGTCTAAAAGGTGT
TAAAATAAACACAAGTGCAGTATTATAATAAGAATGTCTACTTGCAAAATTACATTTAAAC
TTACTTAAAAGTTTTTTATATAGGGTTTCTGCCTTTTCTTTGGTGGTATGTATTACAAATG
CAGTTAAAGTTCTATTACTAAATACAGCTTGAGACACAAATTCTTCCAATTCTTTAGGAAA
TGATAAAGATGCATCTGTAGCATTGTCCTTTTTTTTTTT
>ContigC(06h01.08f04.05h07)
TGGCTTATTAAACACCTGCTTATAAAGCAAATTTATAGTATATGGGTTTCTAACCCTTCT
TTGTCTAAAGTGTAGCCTAAAAAATCTGGAAAGCCCTGTATGTACCCTATTTTTGTTAAC
TCCCAAAAGGTCAGCACAAGTTATGTACAATCTACCTTGCAAGCAAAGAATGCCAATGCC
ATTTTCATCCAACAGTGGAATTGTGCTATTATTACTAAATGACACCACTGGTGGAGTTGC
AGCCCCTCCAACCATTCTGCCAAAGTATCTGCAGTTATCATTGCGGCTAGGGTCTGCCAC
CCATGATTCAATGCTGTACTTTTCATTAGTGACCCTTGCCCTGGGTGTTTCAGAACTGGA
ATAAACATGAGGCACAGTACCATTGGTTTTAGGATTTACAGTATACTGAGCAGGCCCCTT
GTATTTTTCAGTGGGAGCAAGTCCTAGCACATCCAAGGGCTGTCCTCCAACTGCCCAAAA
ATAAAGTTGGGGACCAGCAATACCTCCTACACCCCCTGTAGTAAGTATGCCAGCATTAAG
CACAGGAAGCACCAAAACTTCTGTTTCCATTCTATACAGCTCCCATATAAGCATGTCACA
TTCACTAACCTGATTAGGAATATCAGGGGGAGCAACCTGTGACAAGCTGTAGCACACAGT
AGTATCGGCATCAGGAGTAACATTAGCAGCTTCAGCAGTTTTAAGTGGGCTACTAATAGA
CCAATAATGTGGGGTAGTCCCCTCTGCATTTCCAATAACAGGTTTAACAAACAATTCAA
