# Supplementary material for: Identification of a Novel Polyomavirus from Patients with Acute Respiratory Tract Infections
Source: PLoS Pathog. 2007 May 4;3(5):e64. doi: 10.1371/journal.ppat.0030064 (PMC1864993; doi:10.1371/journal.ppat.0030064)
Supplement: Supplementary file 2 [file ppat.0030064.sg002.pdf]

SV40: 5′GCTCAGAGGCAGAGGCGGCCTCGGCCTCTGCATAAATAAAAAAAATTAGTCAG
CGAGTCTCCGTCTCCGCCGGAGCCGGAGACGTATTTATTTTTTTTAATCAGTC

WU: 5′AAGTTGAGGCTTTTTAGGCCTCAGGCCTCCTTATTATAATAAAAAAAAAGCTAA
TTCAACTCCGAAAAATCCGGAGTCCGGAGGAATAATATTATTTTTTTTTCGATT

5213 5229 1 36

Nucleotide position
