# Supplementary material for: Identification of a Novel Polyomavirus from Patients with Acute Respiratory Tract Infections
Source: PLoS Pathog. 2007 May 4;3(5):e64. doi: 10.1371/journal.ppat.0030064 (PMC1864993; doi:10.1371/journal.ppat.0030064)

Consensus Splice Donor and Acceptor Sites

**Large T Donor**	
Baboon	TAGCTCTGAG GTTGGTTCTG
SV40	TGCAACTGAG GTATTTGCTT
JC	TAGTTCAGAG GTTGGTTGTG
BK	TAGCTCAGAG GTATGTGCTG
**WU**	CTCTTCACAG GTAGAATGCA
**Acceptor**	
Baboon	GTTTTTACAG GTGCCAACCT
SV40	TGTATTTTAG ATTCCAACCT
JC	TTTTTTTTAG GTGCCAACCT
BK	TTTTTTATAG GTGCCAACCT
**WU**	TTTATTATAG ATACCCACAT

**Small T Donor**	
Baboon	AAACTCTAAG GTAACTAAGT
SV40	AAGCTCTAAG GTAAATATAA
JC	AAGCTTTAAG GTAAACCACT
BK	AAGCTTTAAG GTAACTAACT
**WU**	GCTTAACCAG GTAAGCATGT
**Acceptor**	
Baboon	GTTTTTACAG GTGCCAACCT
SV40	TGTATTTTAG ATTCCAACCT
JC	TTTTTTTTAG GTGCCAACCT
BK	TTTTTTATAG GTGCCAACCT
**WU**	TTTATTATAG ATACCCACAT

Putative Small T Antigen Forms

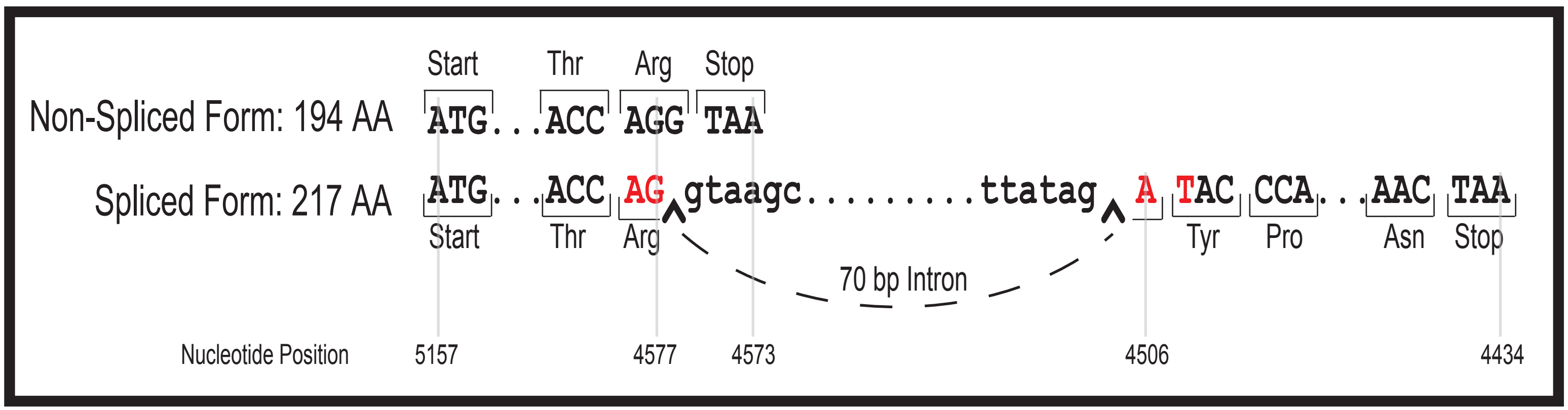

Supplement: Supplementary file 3 [file ppat.0030064.sg003.pdf]
