# Supplementary material for: Identification of a Novel Polyomavirus from Patients with Acute Respiratory Tract Infections
Source: PLoS Pathog. 2007 May 4;3(5):e64. doi: 10.1371/journal.ppat.0030064 (PMC1864993; doi:10.1371/journal.ppat.0030064)
Supplement: Supplementary file 4 [file ppat.0030064.sg004.pdf]

Carboxyl Terminus: Host Range Domain

```
AGMPV   RILNSGYTILLVLLWYNPVDSFTPKVQEKVVQWKETLEKYVSITQFGNIQQNIIDGKDPLHGIVIEEQM----------------------------------------------------------------------------------------------------------------- 697
Hamster RVCQSPLTMLIALLWNVPTENFDKSLKEKVETEKKVLSDMCNFTTFAEMCLNIQRGADPLEAL----------------------------------------------------------------------------------------------------------------------- 751
Murine  RIIQSGDTLALLLIWNFTSDVFDPDIQGLVKEVRDQFASECSYSLFCDILCNVQEGDDPLKDICDIAEYTVY-------------------------------------------------------------------------------------------------------------- 785
Baboon  RILQSGMTLLLLLIWFRPVADFASDIQHRIVQWKERLDSEISMYTFSRMKYNICMGK-CILDWAR-----EEESE-------------TEDSGHGSSTESQSQCFSQASDTSGSADAPASQTPDPYDHDNPYHICKGFVCFKRPKTPPPKCFRQWFGLDLNEEALLWWSHIIGETPFRDLKL 731
BK      RILQSGMTLLLLLIWFRPVADFATDIQSRIVEWKERLDSEISMYTFSRMKYNICMGK-CILDITR-----EEDSE-------------TEDSGHGSSTESQSQCSSQVSDTS--APAEDSQRSDP--HSQELHLCKGFQCFKRPKTPPPK-------------------------------- 695
JC      RILQSGMTLLLLLIWFRPVADFAAAIHERIVQWKERLDLEISMYTFSTMKANVGMGR-PILDFPR-----EEDSE-------------AEDSGHGSSTESQSQCFSQVSEASG-ADTQEN---------CTFHICKGFQCFKKPKTPPPK-------------------------------- 688
SV40    RIIQSGIALLLMLIWYRPVAEFAQSIQSRIVEWKERLDKEFSLSVYQKMKFNVAMGI-GVLDWLRNSDDDDEDSQENADKNEDGGEKNMEDSGHETGIDSQSQGSFQAPQSS--QSVHDHN--------QPYHICRGFTCFKKPPTPPPEPET----------------------------- 708
WU      RVLQSGMCILIMLIWCRPVSDFHPCIQAKVVYWKELLDKYIGLTEFADMQMNVTNGC-NILEKHNA-------------------------------------------------------------------------------------------------------------------- 648
Bovine  RYLQSGITWLLLLIYFRSVDDFTEKLQECVVKWKERIETEVGDMWLLTMKENIEQGK-NILEK----------------------------------------------------------------------------------------------------------------------- 586
MPPV    RILQNGCTLLLLLIYHCDLDDFAESIQGKVRAWKERVNSEISVSTYLEMRQCCLEGRYSVCTKYSN----ANTAQ----------------------------------------------------------------------------------------------------------- 648
BFPV    RLITKPETLLAYLLIRPESEKEISADLRAEFLTVIENLKFEVDERFFQYNNRLHEG-LCVHE------------------------------------------------------------------------------------------------------------------------ 587
Finch   RILTKAETLLALLLINRESEERLSKQVRLDFVTTIDCLKFEVEQRIWKYLYNLHAG-LPYDHED---------------------------------------------------------------------------------------------------------------------- 612
Crow    RLLTRPEVLLGLLILA-DTEVIDKKQISCKSATCVECLKFEFDERWR-YYGKLYEGESCFEQTDT--------------------------------------------------------------------------------------------------------------------- 636
Goose   RWLTKAETLLAMLFLT-TPKSFR-DEITGKCANVLDILKIEFDSRILNYMFRIHNGESCFYEPENTGDI----------------------------------------------------------------------------------------------------------------- 636
ruler   ......770.......780.......790.......800.......810.......820.......830.......840.......850.......860.......870.......880.......890.......900.......910.......920.......930.......940...
```
