# Supplementary material for: Identification of a Novel Polyomavirus from Patients with Acute Respiratory Tract Infections
Source: PLoS Pathog. 2007 May 4;3(5):e64. doi: 10.1371/journal.ppat.0030064 (PMC1864993; doi:10.1371/journal.ppat.0030064)
Supplement: Supplementary file 5 [file ppat.0030064.sg005.pdf]

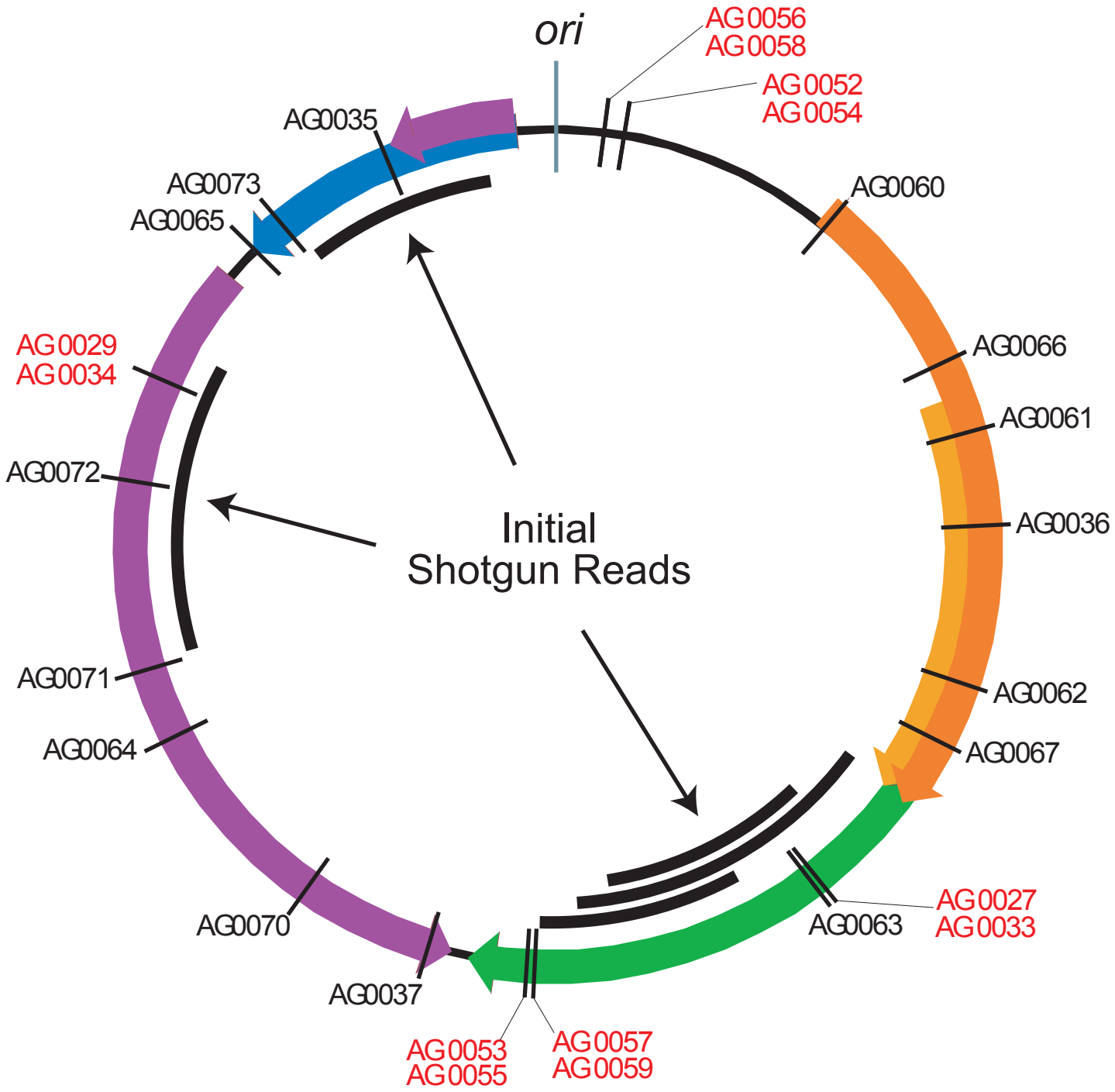
ori
AG0056
AG0058
AG0052
AG0054
AG0060
AG0066
AG0061
AG0036
AG0062
AG0067
AG0027
AG0033
AG0063
AG0057
AG0059
AG0053
AG0055
AG0037
AG0070
AG0064
AG0071
AG0072
AG0029
AG0034
AG0065
AG0073
AG0035
Initial
Shotgun Reads
