# Supplementary material for: Identification of a Novel Polyomavirus from Patients with Acute Respiratory Tract Infections
Source: PLoS Pathog. 2007 May 4;3(5):e64. doi: 10.1371/journal.ppat.0030064 (PMC1864993; doi:10.1371/journal.ppat.0030064)
Supplement: Supplementary file 6 [file ppat.0030064.st001.pdf]

Amplification Primers			Sequencing Primers	
**Name**	**Sequence**	**Size**	**Name**	**Sequence**
AG0027	GCCAGCATTAAGCACAGGA	3043bp	M13F	GTAAAACGACGGCCAG
AG0029	GCTTGAGACACAAATTCTTCCA		M13R	CAGGAAACAGCTATGAC
			AG0035	TGCATTCTACCTGTGAAGAGC
			AG0036	GCATTTACTGGGTCAGATTCC
AG0033	TCCTGTGCTTAATGCTGGC	2229bp	M13F	GTAAAACGACGGCCAG
AG0034	TGGAAGAATTTGTGTCTCAAGC		M13R	CAGGAAACAGCTATGAC
			AG0037	TGCATGTCAGCAAATTCAGT
AG0052	TTATGTGCAGGAATGTGCAG	2552bp	M13F	GTAAAACGACGGCCAG
AG0053	CTCTACTGTGGGAGGCAAGG		M13R	CAGGAAACAGCTATGAC
			AG0066	CAGGATACAATCCCCAAGGA
			AG0067	ACCTTCCTGGTTTTGCTGTG
AG0054	CTGCACATTCCTGCACATAA	2679bp	M13F	GTAAAACGACGGCCAG
AG0055	CCTTGCCTCCCACAGTAGAG		M13R	CAGGAAACAGCTATGAC
			AG0064	ACCAGGCTACACCAGCCATA
			AG0065	ATGCAAGGGGTTGGCTTAAC
AG0056	TAGCAGCCACAAGGTGGAGC	2576bp	M13F	GTAAAACGACGGCCAG
AG0057	AAGGGCCCTGTTTCTTCAGT		M13R	CAGGAAACAGCTATGAC
			AG0060	ATGGGCATATTGCTTGCTGT
			AG0061	TTCATTGCATCCCACCTGCC
			AG0062	ATGCAGCCCCTGACTGGATT
			AG0063	GCTGGCATACTTTACTACAGG
AG0058	GCTCCACCTTGTGGCTGCTA	2655bp	M13F	GTAAAACGACGGCCAG
AG0059	ACTGAAGAAACAGGGCCCTT		M13R	CAGGAAACAGCTATGAC
			AG0070	TGTTACAATACCTGGAGGAA
			AG0071	CCACATCAATAGCCTGTTGA
			AG0072	GCTAATAGTACAGTATCCCT
			AG0073	CAAAGCCAAACCACTCTCTA
